# Sarcopenic obesity in the Asia-Pacific region: Epidemiology, risk factors, and management

**DOI:** 10.1016/j.afos.2025.05.001

**Published:** 2025-05-21

**Authors:** Chun-Feng Huang, Chih-Hsing Wu

**Affiliations:** a Faculty of Medicine, School of Medicine, National Yang Ming Chiao Tung University, Taipei, Taiwan; b Department of Leisure Services Management, Chaoyang University of Technology, Taichung, Taiwan; c Division of Family Medicine, En Chu Kong Hospital, New Taipei City, Taiwan; d Department of Family Medicine, National Cheng Kung University Hospital, College of Medicine, National Cheng Kung University, Tainan, Taiwan; e Department of Family Medicine, College of Medicine, National Cheng Kung University, Tainan, Taiwan; f Institute of Gerontology, College of Medicine, National Cheng Kung University, Tainan, Taiwan

**Keywords:** Sarcopenic obesity, Asia-Pacific, Lifestyle intervention, Public health

## Abstract

Sarcopenic obesity (SO), characterized by the concurrent presence of sarcopenia and obesity, is an emerging public health challenge in the Asia–Pacific region. With rapid population aging and increasing obesity rates, the prevalence of SO is increasing, particularly among individuals over 60 years of age. This condition results from a complex interplay of muscle loss, fat accumulation, chronic inflammation, hormonal changes, and metabolic dysregulation, leading to heightened risks of frailty, disability, cardiovascular disease, and mortality.

Region-specific risk factors, including dietary transitions, reduced physical activity, and socioeconomic disparities, further contribute to its increasing prevalence. While pharmacological options are under investigation, lifestyle modifications remain the cornerstone of prevention and management. Regular resistance training, adequate protein intake, and balanced nutrition are essential for preserving muscle mass while promoting fat reduction. Community-based interventions, such as structured exercise programs, public health campaigns, and urban planning that encourage active aging, are crucial for sustainable long-term outcomes.

The current inconsistency in diagnostic criteria has led to numerous challenges, highlighting the urgent need for consensus. In addition, targeted policies focusing on nutritional education, elderly friendly infrastructure, and access to preventive healthcare are essential to alleviating the burden of SO. A comprehensive approach that integrates lifestyle interventions, clinical advancements, and supportive policies is crucial for effectively addressing the growing impact of SO in the Asia–Pacific region and improving health outcomes for the aging population.

## 1 Introduction

Sarcopenic obesity (SO) is characterized by the simultaneous presence of sarcopenia, which refers to the age-related decline in muscular mass, strength, and function, and obesity, which is defined as an excess of adipose tissue, in a single individual [1]. The World Health Organization characterizes obesity in East Asians as a body mass index (BMI) of ≥25 kg/m^2^, but previous studies categorized obesity on the basis of a body fat mass/body weight above 27% in males and 35% in females [1,2]. Approximately 11% of older adults worldwide are estimated to have SO, with a significant increase observed after the age of 70 [2]. This syndrome is becoming an increasing public health issue in the Asia-Pacific region and is characterized by a rapidly aging population and a rising prevalence of obesity [3]. Sarcopenic obesity involves a synergistic interaction between its two components, intensifying health risks and resulting in a detrimental cycle of muscle loss and fat accumulation. Compared with individuals with either sarcopenia or obesity alone, older adults with SO face increased risks of frailty, cardiovascular disease, fractures, disability, and mortality [4].

The importance of SO in the Asia–Pacific region is highlighted by demographic shifts [3,5]. By 2050, one in four individuals in the Asia–Pacific region will exceed 60 years of age, with the “oldest-old” (≥ 80 years) representing approximately one-fifth of the total elderly population [6]. This extraordinary aging, along with lifestyle changes and increasing obesity, exerts significant pressure on healthcare systems. Inadequate intervention in SO can increase hospitalizations, necessitate long-term care, and increase healthcare expenditures. Identifying and mitigating SO is essential for promoting good aging and ensuring sustainable healthcare in the Asia-Pacific region [7].

There is an urgent need to increase the identification and management of SO in the Asia–Pacific region. This review seeks to clarify the epidemiology of SO by utilizing country-specific data from the Asia–Pacific region, outlining the underlying pathophysiological mechanisms, evaluating region-specific risk factors and screening debates, and consolidating current evidence on both nonpharmacological and pharmacological treatment modalities.

## 2 Epidemiology

### 2.1 Prevalence in Asia–Pacific countries

SO is increasingly acknowledged as a significant health issue in the Asia-Pacific region, with prevalence rates differing markedly among countries due to variations in aging demography, dietary practices, and levels of physical activity (Table 1). The disorder is more common in older people, especially those over 60 years of age, and is linked to heightened risks of frailty, disability, and chronic illness. Recent research indicates that the prevalence of SO in the Asia‒Pacific region ranges from 5% to 20%, although variances in diagnostic criteria result in disparities in reported rates [8].

**Table 1:** Study findings of sarcopenic obesity in the Asia–Pacific region.

Country/Region	Guideline/Study	Population	Age Group	Diagnosis	Prevalence	Reference
China	2024 Multicenter Study	Men and women	≥60 (subgroups: 70–79, ≥80)	ASM + handgrip; BMI/body fat %	3.58% (M), 2.88% (F) 70–79: 6.58% (M), 4.40% (F) ≥80: 13.16% (M), 18.18% (F)	Yang et al., 2024 [11]
Japan	Japanese Working Group (2024)	Rehabilitation patients	Elderly	Stepwise: BMI, WC, calf circumference + handgrip, muscle mass	4.3%–5.3%	Ishii et al., 2024 [17]
South Korea	KNHANES IV + community studies	Men and women	65–74, 75–84, ≥60	AWGS-aligned + BMI	6.1% (M), 7.3% (F) 75–84: 15.46% (M), 13.59% (F) 65–74: ∼9.09% (M)	Hwang et al., 2012 [15]; Hwang & Park, 2024 [5]
India	SO-CUBES	Healthy older adults	≥65	BMI, WC, or body fat %	5.4%–6.3%	Pal et al., 2021 [20]
Taiwan	AWGS 2019 + Taichung Declaration	Older adults	Unspecified	AWGS 2019-based + BMI or body fat %	Not specified	Chen et al., 2020 [22]; Taichung Declaration [23]
Southeast Asia (Singapore, Malaysia, Thailand)	AWGS framework	Community-dwelling older adults	Unspecified	Same as AWGS 2019	Not specified	Chen et al., 2020 [22]

AWGS, Asian Working Group for Sarcopenia; BMI, body mass index; KNHANES: Korea National Health and Nutrition Examination Survey; SO, sarcopenic obesity; WC, waist circumference.

Countries in the Asia–Pacific region demonstrate distinct patterns of SO prevalence, shaped by genetic, socioeconomic, and public health policy factors. Urbanization, the adoption of Western eating trends, and declining physical activity levels have all led to increasing SO rates in numerous countries. Conversely, several rural cultures present reduced prevalence rates attributable to traditional lifestyles characterized by increased physical activity and diminished obesity levels. The transition to a Westernized diet and a sedentary lifestyle has led to an increase in the prevalence of sarcopenia and obesity [3,8]. Compared with their Western counterparts, Asian populations typically have a lower BMI but greater fat mass at equivalent BMI levels, hence confounding the detection of SO [9], [10].

The incidence of SO in Chinese communities among elderly people is generally modest, although it significantly increases with increasing age. A 2024 multicenter survey (N = 2821) reported an overall prevalence of SO of 3.58% in men and 2.88% in women [11]. The prevalence increased to 6.58% for males and 4.40% for women in the 70–79 years age group and then increased to 13.16% in men and 18.18% in women aged 80 years and older. SO is associated with an increased risk of cardiovascular disease. SO has been linked with elevated risks of cardiovascular disease (HR = 1.39), heart disease (HR = 1.36), and stroke (HR = 1.40) compared with the optimal reference group, highlighting the health burden in this demographic [12]. Japan has commenced initiatives to define and measure SO in response to its aging demographic. A study employing newly established diagnostic criteria reported a prevalence of SO of approximately 4.3%–5.3% among elderly Japanese rehabilitation patients [13,14]. In Korea, the Fourth Korea National Health and Nutrition Examination Survey (KNHANES IV) reported SO prevalence rates of 6.1% in men and 7.3% in women over the age of 60 [15]. Conversely, Southeast Asian countries such as India demonstrate somewhat lower prevalence rates due to diminished obesity levels [16].

Japan has initiated efforts to delineate and quantify SO in light of its aging population. The Japanese Working Group on Sarcopenic Obesity has developed specific diagnostic criteria for their population, recognizing ethnic variations in body composition [17]. The criteria utilize a two-step methodology: (a) initial screening using Japanese thresholds (eg, BMI ≥ 25 kg/m^2^; waist circumference (WC) ≥ 85 cm for men/ ≥ 90 cm for women) and basic sarcopenia assessments (eg, the Yubi-Wakka “finger-ring” test, to identify older adults with low muscle mass, calf circumference < 34 cm and < 33 cm for men and women, respectively), followed by (b) a diagnosis that verifies diminished handgrip strength, prolonged chair-stand duration, decreased limb muscle mass (adjusted for BMI), and elevated visceral fat area or body fat percentage. According to these criteria, Stage I SO is characterized by muscle weakness or low function, accompanied by diminished muscle mass and obesity, whereas Stage II SO encompasses these elements in addition to associated comorbidities [17]. Although accurate nationwide prevalence rates based on these new criteria are under validation, they highlight Japan's acknowledgment of SO as a significant geriatric syndrome.

The swift aging of Korea's population, which is anticipated to reach 40% of the population aged 65 and older by 2050, has necessitated comprehensive monitoring of SO across the nation [5]. A community-based study of persons aged 75–84 years reported a prevalence of SO of 15.46% in males and 13.59% in females, utilizing criteria consistent with Asian guidelines [18]. An additional examination of “young-old” guys aged 65–74 years revealed a weighted prevalence of SO of approximately 9.09%, indicating that approximately 1 in 11 younger seniors experienced SO [5]. The Korean estimates are somewhat elevated compared with those from China or Japan, possibly because of varying diagnostic thresholds or demographic characteristics. In accordance with prevailing trends, the prevalence of obesity among older persons in Korea increased markedly from 2009 to 2019 (eg, men in their 70s: 34.1%–40.2%; women: 37.6%–42.9%), which likely contributed to the increasing incidence of SO cases [19].

Data from other Asia–Pacific nations are limited but expanding. A study conducted in Chandigarh, India, revealed that the prevalence of SO among healthy old individuals (≥ 65) ranged from 5.4% to 6.3% on the basis of BMI, WC, or body fat percentage [20]. This is significant in a South Asian context where undernutrition has historically prevailed; the simultaneous presence of obesity and sarcopenia underscores an epidemiological shift [21]. Singapore, Thailand, Malaysia, and Taiwan have commenced research within the framework of the Asian Working Group for Sarcopenia (AWGS) [22]. Furthermore, the Taichung Declaration for Sarcopenic Obesity 2024, adopted in Taiwan during the International Conference on Sarcopenic Obesity, emphasizes the serious health risks associated with SO. The Asia-Oceania Association for the Study of Obesity (AOASO) and the International Association of Gerontology and Geriatrics Asia/Oceania Region (IAGG-AOR) promote preventive measures and improved healthcare systems for SO [23]. However, a prevalent trend is the escalation of SO with increasing age, resulting in heightened rates of older demographics (80+ years), which poses a significant concern for the Asia–Pacific region, where the oldest-old population is developing most rapidly. Efforts to define and declare SO operations in the Asia–Pacific region highlight the need for intervention in this area.

### 2.2 Trends over time and regional comparison

Trends in the Asia–Pacific region reveal a concurrent rise in SO alongside an aging population and increasing obesity rates. Countries such as Japan and Korea, which underwent early and swift demographic aging, were among the first in Asia to document sarcopenia and subsequently SO. Korea's data from 2008 to 2019 indicate increasing obesity rates among seniors, suggesting a higher incidence of SO in current cohorts than in previous cohorts [5,19]. China's continuous urbanization and nutritional shift have resulted in increased adult obesity rates, leading to a rise in concomitant sarcopenia among elderly individuals who experienced periods of scarcity [11]. Southeast Asian nations shifting from undernutrition to overnutrition are beginning to see SO as a burgeoning concern among their elderly population [24,25].

In comparison, the prevalence of SO in the Asia–Pacific region is typically lower than that in Western nations when Western obesity thresholds are applied, as Asian populations frequently exhibit lower BMIs yet more body fat at a specific BMI [26]. Nevertheless, when Asian-specific criteria (eg, BMI ≥27 kg/m^2^ for obesity) are employed, the disparity diminishes. Certain Western studies indicate that the prevalence of SO is approximately equal among older adults, which corresponds to the upper range of Asia–Pacific findings [2]. A significant distinction is that Asia–Pacific criteria for obesity account for elevated body fat at reduced BMI levels; for example, the WHO advises a BMI of ≥27 kg/m^2^ for obesity in Asians compared with 30 for Western populations, whereas waist circumference thresholds are lower (approximately 90 cm for Asian males versus 102 cm for Western men) [27]. Consequently, prevalence estimates may differ significantly according to the criterion employed. A Chinese study demonstrated that, among the same older persons, the prevalence of obesity varied from 4.1% to 42.2% according to the measurement used—BMI, WC, body fat percentage, or visceral fat area—while the prevalence of SO ranged from 0.1% to 7.9% [28]. The absence of a standardized definition complicates direct comparisons.

Nevertheless, the prevailing trend is an increase in SO throughout the Asia–Pacific region. As younger generations with elevated obesity rates resulting from contemporary sedentary lifestyles and dietary habits advance into old age, the interplay of obesity and sarcopenia is anticipated to become increasingly common [10]. Epidemiological surveillance, encompassing longitudinal investigations, remains essential in numerous Asia–Pacific nations to assess temporal patterns. Presently, regional collaborative initiatives such as the AWGS and nation-specific working groups (eg, Japan's Working Group on Sarcopenic Obesity) are engaged in standardizing criteria, collecting data, and enhancing awareness [17].

## 3 Pathophysiology

Sarcopenic obesity results from a complex interaction of molecular pathways in which obesity-related processes hasten muscle loss, whereas sarcopenia-related alterations intensify fat storage. Essential pathophysiological elements include the following.

### 3.1 Muscle atrophy and diminished regeneration

The aging process results in a gradual decline in muscle fibers and motor units [29]. Sarcopenia is characterized by diminished muscle protein synthesis resulting from anabolic resistance, decreased hormonal support (such as growth hormone, testosterone, and IGF-1), and malfunction of satellite cells [30]. Obesity can exacerbate this condition by encouraging inactivity and potentially by intramuscular fat infiltration (myosteatosis), which diminishes muscle quality and contractility [31]. A prolonged positive energy balance without sufficient protein or resistance training results in a decline in muscle protein turnover [32]. Additionally, adipose tissue can release substances, such as adipokines, that impede myogenesis, and obesity is correlated with increased levels of myostatin, a muscle growth inhibitor [33,34]. Consequently, both age and fat lead to muscular atrophy.

### 3.2 Chronic low-grade inflammation

A key factor between fat and sarcopenia is inflammation. In obesity, the proliferation of adipose tissue accommodates elevated levels of proinflammatory immune cells (M1 macrophages, T cells) that release cytokines (TNF-α, IL-6, and IL-1β) [35,36]. These cytokines can trigger muscle protein degradation and hinder muscle synthesis, accelerating sarcopenia. Aging is concurrently linked to “inflammaging,” a persistent low-grade inflammatory condition [37]. The intersection of obesity-related and age-associated inflammation fosters an environment that accelerates muscle breakdown and inhibits anabolic signaling. Research on SO in the Asia–Pacific region revealed elevated inflammatory markers and oxidative stress in muscle and adipose tissues, highlighting the harmful loop of inflammation-driven tissue destruction [38].

### 3.3 Hormonal and endocrine modifications

Obesity and aging affect essential hormones. In elderly individuals, the levels of anabolic hormones, including testosterone, growth hormone, and dehydroepiandrosterone (DHEA), decrease, hence attenuating muscle-building signals [39,40]. Obesity exacerbates testosterone deficiency in men and may induce insulin resistance; elevated insulin and insulin-like growth factor 1 (IGF-1) initially facilitate fat accumulation, but prolonged insulin resistance adversely affects muscle protein metabolism [41]. Adipose tissue secretes excess leptin, which may diminish appetite regulation in the brain while promoting inflammation peripherally, and decreases adiponectin, an anti-inflammatory adipokine that offers protection [42]. The overall outcome is a hormonal milieu conducive to adiposity and muscular atrophy. Low free testosterone levels are correlated with an increased incidence of SO in males. Imbalances in thyroid hormones and increased cortisol levels due to chronic stress or Cushingoid conditions may also contribute [43]. Moreover, vitamin D deficiency, which is prevalent among elderly and obese adults, may hinder muscle function and regeneration [44].

### 3.4 Metabolic dysregulation

Sarcopenic obesity frequently coexists with the characteristics of metabolic syndrome, including insulin resistance, dyslipidemia, and hypertension [45,46]. Excess adiposity, particularly visceral fat, results in ectopic fat deposition in the liver and muscle, causing lipotoxicity that harms mitochondria and muscle fibers [47]. Mitochondrial failure in muscle, resulting from aging and fat infiltration, diminishes ATP production, exercise ability, and recovery, hence fostering increased sedentary behavior and muscular atrophy [48]. The ability of obesity to decrease the metabolic rate per unit of body mass, due to the lower metabolic activity of fat than of muscle, results in weight gain that further diminishes relative energy expenditure, exacerbating obesity. A reduction in muscle mass diminishes the basal metabolic rate and glucose clearance, resulting in insulin resistance that promotes fat accumulation [49,50]. This generates a feedback loop: obesity-related metabolic dysfunction hastens sarcopenia, whereas sarcopenia-related metabolic decline worsens obesity.

### 3.5 Fibrosis and quality of tissue

Both adipose expansion and muscle atrophy involve tissue remodeling [51]. In obesity, adipose tissue frequently undergoes fibrosis and hypoxia as it increases, resulting in inflammation [52]. Sarcopenia is defined by the buildup of fibrous connective tissue and adipose tissue inside muscle, resulting in reduced muscular quality [29,53]. The qualitative changes imply that, even with relatively low muscle mass, the functional decline is more significant, with strength loss outweighing mass loss. The infiltration of muscle, including intermuscular and intramuscular fat, by adipose tissue is strongly related to impaired muscle function in SO.

### 3.6 Shared molecular pathways

Research indicates that specific signaling pathways are critical in SO. Akt/mTOR signaling (an anabolic pathway) is frequently inhibited by both aging and fatty acids/inflammation [54]. Cytokines and oxidative stress stimulate the NF-κB and FOXO pathways, which promote atrophy and catabolism, respectively. Adipose-derived factors such as FGF21 and GDF15, which cause obesity, may also influence muscle metabolism [55]. Myokines (muscle-derived cytokines), such as irisin or interleukin-15, can influence fat, and their dysregulation may link muscle and fat crosstalk [56].

**Fig. 1:**
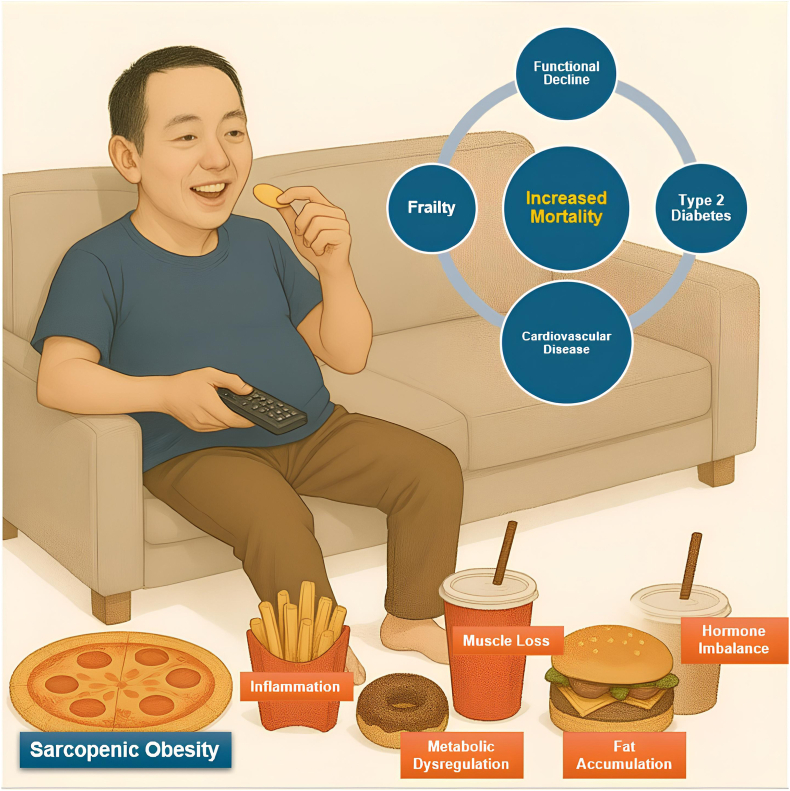
Risk factors and consequences of sarcopenic obesity in illustration.

The etiology of SO is multifaceted and characterized by a detrimental cycle of physical inactivity, inadequate nutrition, and biological aging. An unhealthy diet and sedentary lifestyle lead to excess adiposity (elevating inflammatory cytokines, leptin, and insulin resistance) while concurrently causing muscular disuse (reducing muscle protein synthesis and anabolic hormones) [10]. This results in a state of SO, marked by elevated proinflammatory cytokines, modified myokines (eg, increased myostatin), mitochondrial dysfunction in muscle, and anabolic resistance. Figure 1 depicts the probable risk factors and consequent effects of SO in illustration. The interaction between muscle and fat in SO is a prominent research focus, with current studies seeking to elucidate mechanisms such as immunosenescence, gut microbiome effects, and genetic predispositions [57,58].

## 4 Risk factors

Sarcopenic obesity has a multifaceted etiology with contributions from genetic, lifestyle, dietary, and socioeconomic factors. In the Asia–Pacific context, several region-specific risk factors have been identified or are under exploration.

### 4.1 Age and sex

Age is the strongest risk factor, as sarcopenia is age-related by definition [29]. The risk accelerates after 60 years of age and even more after 75–80 years of age. Women, especially postmenopausal women, often have greater body fat at a given BMI and experience hormonal changes (estrogen loss) that can hasten sarcopenia [59]. However, data on sex differences in SO incidence are mixed [18]. Some Asian studies reported greater SO in men than in women, possibly due to men's greater tendency toward central obesity and greater absolute muscle loss. Other studies have reported increased SO in women due to longevity and increased obesity rates in older women. For example, a Chinese study revealed no significant difference in the prevalence of sarcopenia between men and women. In contrast, the National Health and Nutrition Examination Surveys 1988–1994 looked at 1526 women and 1391 men over the age of 70 [60]. To determine body fat percentage, the prediction method evaluated waist and hip circumference, triceps skinfolds, and sex. Height, age, bioelectrical impedance analysis resistance, and sex were utilized to predict muscle mass. Individuals with > 40% fat mass and < 40% muscle mass were classified as SO. The SO rate was 7.4% in women and 9.6% in men [61]. In the Asia-Pacific region, where women often live longer but may face lifelong nutritional disparities, both sexes are at risk, but risk profiles differ [62].

### 4.2 Genetic and ethnic predisposition

Genetic factors influencing body composition (propensity for visceral vs subcutaneous fat, muscle fiber type distribution, etc.) could affect SO risk [63]. Certain gene polymorphisms (eg, in TNF-α, IL-6, or muscle growth regulators such as myostatin) have been implicated in sarcopenia and obesity separately [64]. Research on gene variants in Asian populations is emerging; for example, an Iranian study (not Asia–Pacific but relevant) suggested that a TP53 polymorphism was associated with increased SO risk [65]. In East Asians, certain alleles related to type II diabetes and obesity (such as the FTO gene) could indirectly increase SO risk via obesity [66]. Ethnic-specific body composition differences are notable: Asians generally have more body fat at lower BMIs than Europeans do, which may predispose them to “normal-weight obesity” and SO in later years [67]. Moreover, the “thrifty phenotype” hypothesis suggests that undernutrition in early life (common in older Asia–Pacific generations) followed by caloric surplus in adulthood leads to greater central fat and muscle insulin resistance—a recipe for SO [68]. Genetic predispositions for early sarcopenia (eg, differences in muscle fiber loss rates) combined with genetic risks for obesity (eg, appetite regulation) could synergize in certain populations.

### 4.3 Lifestyle and physical activity

Sedentary behavior is an important modifiable risk factor for SO [69]. Physical inactivity causes muscular atrophy and lower energy expenditure, which promotes fat growth. Asian urbanization has reduced daily physical activity; for example, replacing agrarian lifestyles with office work and screen time has increased sedentariness. A study indicated that reduced daily steps were associated with SO in nearly 50% of treatment-seeking obese adults [70]. In many Asia–Pacific cities, older adults may have limited space for exercise or face environmental barriers (pollution, traffic) to stay active. Culturally, retirement often leads to decreased activity. Conversely, societies with ingrained habits of tai chi, yoga, or community exercises might mitigate risk [71].

### 4.4 Diet and nutrition

Dietary patterns in the Asia-Pacific region have shifted toward higher-calorie, nutrient-poor foods. Insufficient protein intake in older adults is a known risk factor for sarcopenia [72]. Traditional Asian diets are plant heavy and relatively low in protein; however, many elderly individuals may not consume the recommended amount of protein (1.0–1.2 g/kg/day) for muscle maintenance. Evidence-based guidelines suggest that older people need more protein than younger adults do for anabolism, and intakes <0.8 g/kg are linked to greater health decline [73]. Asian cuisines vary, but vegetarian practices or simple low protein affordability can lead to chronic protein insufficiency. Vitamin D deficiency is prevalent in South Asia and East Asia due to low sun exposure (especially in veiled cultures or highly urban societies) and a lack of food; vitamin D deficiency is critical for muscle function, and its lack is associated with sarcopenia [44,74]. Diets high in refined carbohydrates and fats contribute to obesity without providing the amino acids needed to preserve muscle. Additionally, high sodium and low fruit/vegetable intake can contribute to hypertension and metabolic syndrome, which are correlated with SO [75]. Conversely, caloric restriction without adequate protein can cause weight loss with disproportionate muscle loss; many older Asians (particularly women) might unintentionally worsen sarcopenia if trying to lose weight without guidance [76].

### 4.5 Socioeconomic factors

Socioeconomic status (SES) influences risk through nutrition and health behaviors in developing parts of Asia, poverty during childhood can cause stunting and low muscle mass accrual, and economic improvement can lead to obesity in midlife—a combination that predisposes individuals to SO later [77]. Elderly individuals with low SES may have food insecurity, leading to a reliance on cheap, high-calorie foods but insufficient protein (the “double burden” of malnutrition) [72]. Education level can affect awareness of healthy lifestyles; individuals with less education might be less aware of sarcopenia or how to exercise safely in old age [78]. Additionally, urban vs rural differences exist. Urban older adults may have better access to healthcare (and thus sarcopenia screening, gyms, or parks) but also greater obesity from sedentary lifestyles; rural elderly individuals may remain more active physically but could have poorer diet quality [71]. Asian cultures that revere elderly individuals might provide them with better care, but in some cases, older persons live alone or in neglected conditions, exacerbating malnutrition and physical inactivity [79].

### 4.6 Chronic diseases and medications

The high prevalence of type 2 diabetes and metabolic syndrome in Asia is a risk factor for SO [80]. Diabetes causes muscle protein breakdown (due to insulin resistance) and often coexists with obesity [81]. Moreover, complications such as peripheral neuropathy reduce physical activity, furthering muscle loss [82]. Other chronic diseases common in aging (heart failure, chronic obstructive pulmonary disease, arthritis) can lead to cachexia or reduced mobility, which, when combined with obesity (especially if the person intentionally or unintentionally gains weight earlier in life), can result in SO [83]. Certain medications can also contribute: long-term steroids (for chronic obstructive pulmonary disease or rheumatoid arthritis) cause muscle atrophy and fat redistribution; some diabetes medications cause weight gain; and beta-blockers might reduce exercise capacity [84].

In summary, the risk factors in the Asia–Pacific region encompass the intersection of rapid lifestyle changes and traditional challenges. Genetic predispositions and early-life undernutrition set the stage, whereas modern sedentariness and diets high in calories but low in protein quality drive the condition in later years. Recognizing these factors is vital for targeted management. For example, interventions may emphasize ensuring sufficient protein and micronutrient consumption in older people, advocating culturally appropriate activity, and incorporating sarcopenia screening into chronic disease treatment, such as diabetes or heart failure. Socioeconomic support, such as food programs for the elderly or “age-friendly cities” that encourage physical activity, can also mitigate the risk of SO at the population level.

## 5 Screening

Screening for SO in the Asia–Pacific region is complex because of the need to assess both muscle mass and adiposity. Despite the absence of a universally accepted definition, regional criteria have been established. The AWGS 2019 provides sarcopenia cutoffs tailored to Asian populations, defining low muscle strength, mass, and physical performance [22]. It also introduced “possible sarcopenia” for early community screening. For obesity, a BMI ≥25 kg/m^2^ is often used, but the criteria vary by country. The ESPEN/EASO 2022 consensus recommends a two-step screening strategy for SO. The first step involves initial risk assessment using BMI, waist circumference, SARC-F, or calf circumference, followed by confirmation through muscle function tests such as grip strength, gait speed, or the chair stand test. The SARC-F questionnaire is a quick screening tool for assessing sarcopenia risk, strength, walking ability, rising from a chair, stair climbing, and fall history through five questions. Many elderly care facilities and community screening programs use the SARC-F as an initial assessment. If a respondent scores above the threshold (eg, ≥ 4 points), further detailed evaluation is recommended. Diagnosis requires both impaired muscle function and low muscle mass, assessed as appendicular lean mass (ALM/height^2^) via dual-energy X-ray absorptiometry (DXA) or bioelectrical impedance analysis (BIA) [1]. Both the AWGS 2019 and ESPEN/EASO 2022 consensus emphasize a standardized screening process to improve early detection and intervention. Japan's 2024 consensus follows a similar approach, incorporating the “Yubi-Wakka” (finger-ring) test for muscle mass screening [17]. Physical function tests, including handgrip strength tests, chair-stand tests, and gait speed assessments, are commonly integrated into community programs, particularly in Hong Kong and Taiwan [22,85].

To date, the major challenge is a lack of defined diagnostic criteria, which leads to underdiagnosis, particularly when healthcare providers rely solely on BMI, ignoring normal-weight obesity with increased fat levels. Given time constraints in primary care, AWGS's “possible sarcopenia” concept allows for early lifestyle modifications without requiring a full body composition analysis. The adoption of a two-step screening consensus is crucial for increasing SO detection and management in the Asia-Pacific region.

## 6 Intervention

Management of SO in the Asia–Pacific region requires a multimodal approach, integrating nonpharmacological interventions (lifestyle modifications such as diet and exercise) as the first line and pharmacological options when appropriate. Region-specific considerations, such as cultural diet patterns and available medications, also play a role.

### 6.1 Non-pharmacological interventions

#### 6.1.1 Dietary strategies

Nutrition intervention is pivotal. The goal is to achieve fat loss while preserving or augmenting muscle mass, which requires an energy deficit with adequate protein and micronutrients.

##### 6.1.1.1 Caloric restriction with adequate protein

A moderate caloric deficit (typically 250–500 kcal/day) is recommended for weight loss in obese older adults, but pure dieting can exacerbate muscle loss if protein is insufficient [86]. Studies emphasize a high-protein diet (≥1.2 g/kg/day) during weight loss to preserve muscle. For example, a meta-analysis showed that combining energy restriction with exercise was superior in preserving fat-free mass than diet alone [87]. Another meta-analysis specifically in SO patients revealed that protein supplementation plus exercise led to improvements in muscle mass and function [88]. Therefore, many guidelines advise older Asians to consume at least 1.0–1.2 g/kg protein, possibly more (up to 1.5 g/kg) if they are frail or have acute illnesses [89,90]. The exception applies to individuals with stage IV chronic kidney disease (estimated glomerular filtration rate < 30 mL/min/1.73 m^2^), where clinical judgment should be exercised [91]. In practice, this may involve incorporating regionally common protein-rich foods such as fish, eggs, soy products, and dairy (if tolerated). For example, Japanese diets can leverage fish and soy; Indian diets can emphasize dal (lentils) and dairy; and Southeast Asians might use eggs and fish. Whey protein or soy protein supplements are options if whole food intake is insufficient. Notably, a Japanese rehab study suggested that obesity in SO should be defined by body fat % > 30% (men) and > 35% (women), highlighting the importance of fat loss rather than just weight; diet must target fat reduction [92].

##### 6.1.1.2 Protein timing and leucine

Ensuring that proteins are evenly distributed and leucine-rich might maximize muscle protein synthesis in older adults who have anabolic resistance [89]. The incorporation of leucine-rich foods (dairy, soy, meats, and fish) or supplements (such as essential amino acids) has been noted to be helpful. In Asia, a practical tip involves adding milk powder or soy milk to breakfast, having fish or lean meat with lunch, and a protein snack (like yogurt or nuts) in the evening [93].

##### 6.1.1.3 Micronutrients

Addressing deficiencies is important. Vitamin D supplementation is often needed, as deficiency is widespread in older Asians [94]. Combined vitamin D (eg, 800–1000 IU/day) and calcium help bone and muscle; vitamin D plus resistance exercise has shown additive benefits in muscle strength [95]. Additionally, antioxidants (from fruits/vegetables or supplements) might counteract some oxidative stress in muscle, although the evidence is mixed [96,97]. Omega-3 fatty acids (fish oil) have some anabolic and anti-inflammatory effects that could be useful; fish, which are common in many Asian diets, are helpful [98]. A multinutrient approach was used in a trial where resistance exercise + nutritional supplementation (vitamin D and protein) outperformed either alone [99].

##### 6.1.1.4 Dietary patterns

Encouraging a balanced diet such as a high-protein, low-fat, or moderate-carb diet is common. A Mediterranean-style diet (rich in fruits, veg., lean protein, and healthy fats) has evidence for sarcopenia prevention but may need localization [100]. For example, a “Japanese diet” high in fish, soy, vegetables, and adequate protein can be modeled, or a “vegetarian Indian diet” can be optimized by adding dairy and legumes for protein. The avoidance of excessive refined carbs and sugary drinks (which add fat mass) is emphasized [101]. Hydration and fiber are also important for overall health and for enabling exercise [102].

##### 6.1.1.5 Caloric sufficiency if underweight

In some cases of SO (especially “normal-weight obesity”), an older adult might not appear obese by weight but has high fat and low muscle. If they are actually underweight or normal weight, the focus might be on muscle gain rather than weight loss by providing adequate calories and protein while minimizing fat gain [103]. This nuance is important in Asia, where BMI may not be high but body fat is.

#### 6.1.2 Physical activity programs

Exercise is the cornerstone to counter muscle loss and improve fat metabolism. The best results come from combining resistance training (to build/maintain muscle) and aerobic exercise (to burn fat and improve cardiovascular health).

##### 6.1.2.1 Resistance training

Numerous studies, including meta-analyses, have shown that progressive resistance training increases muscle strength and can increase lean mass even in older individuals [104]. For SO, resistance exercise is vital to prevent further muscle loss during weight loss [105]. Programs typically involve 2–3 days per week of major muscle group exercises. In Asia, community centers may lead group strength exercises (using resistance bands, body weight exercises such as chair stands, wall push-ups, and squats). Even using homemade weights (water bottles) or tai chi (which has some strength component) can be beneficial. A meta-analysis cited in a review revealed that resistance exercise plus protein supplementation had beneficial effects on muscle mass and function [106]. Ensuring supervision or guidance is key, as many older adults are unfamiliar with resistance training [107]. Countries such as Japan and China have senior exercise programs, eg, “Rajio Taiso” in Japan (radio calisthenics) and community qigong in China—while not heavy resistance, these programs improve function and serve as gateways to more structured training [108,109].

##### 6.1.2.2 Aerobic exercise

Activities such as walking, cycling, swimming, or even dancing improve cardiovascular fitness and help create an energy deficit for fat loss [110]. The general recommendation is at least 150 min of moderate aerobic activity weekly. In SO, starting slowly is fine (even 10-min brisk walks). Programs in Asia often incorporate cultural forms, such as walking groups in parks (ubiquitous in China at dawn), group dance exercises in community halls, or walking on temple grounds. Aerobic exercise also helps improve insulin sensitivity and reduce visceral fat, which is particularly important in Asians, who tend to accumulate visceral fat [111].

##### 6.1.2.3 Lifestyle modification

Dietary modifications and regular physical activity (with emphasis on aerobic and resistance training) may be the most effective therapies for managing SO [112]. One meta-analysis revealed that only the combination of exercise + nutritional intervention significantly increased muscle mass in SO patients, whereas exercise alone did not [113]. This demonstrates synergy: nutrition offers building blocks and an optimal hormonal environment, whereas exercise stimulates muscle growth and fat burn. Public health initiatives in Asia are increasingly promoting such combined programs. For example, Taiwan's sarcopenia prevention programs for older adults emphasize balanced nutrition, adequate physical activity, and minimizing sedentary behavior [114]. In rural communities, integrating exercise into daily routines (such as squatting while gardening and carrying groceries as weight training) is also advocated. In addition to engaging in formal exercise, reducing sedentary time (eg, avoiding sitting >1 h without standing up) and increasing daily activities (taking stairs, doing housework) contribute significantly. In many Asia–Pacific cultures, the family plays a role; thus, educating families to involve elders in activities (such as playing with grandchildren and short walks) helps. Balance and flexibility exercises (yoga, tai chi) can be complemented by reducing fall risk and improving mobility, indirectly supporting continued exercise engagement [115].

##### 6.1.2.4 Community and policy initiatives

Some region-specific programs include Japan's nationwide frailty prevention classes, China's “National Fitness” plan, which includes elderly fitness corners in parks, and Australia's Stepping On program, which includes strength and balance for seniors. A notable public health strategy is building age-friendly environments—safe walking paths, exercise equipment in parks (commonly seen in Taiwan, Korea, and Singapore), and senior exercise groups [[116], [117], [118]]. Such policies encourage routine physical activity among older adults, which is critical for managing SO at the population level.

To summarize, controlling SO involves a delicate balance. Although obese seniors benefit from weight loss (which improves their metabolic profile and mobility), it should be performed gradually, and muscle-preserving measures should be used. Research indicates that modest weight loss (∼5%–10% over 6–12 months) paired with exercise improves physical function without causing muscle loss [119]. In Asia, older people may be hesitant to lose weight for fear of weakness; consequently, education is required to ensure that weight loss, when performed correctly (with protein and exercise), targets fat and improves strength relative to body weight. An encouraging study revealed that most older people who intentionally lose weight should not develop substantial sarcopenia if they engage in enough exercise and protein [120].

### 6.2 Pharmacological interventions

Currently, there are no medications specifically approved for SO, and effective pharmacotherapy remains limited. However, certain pharmacological approaches target obesity or sarcopenia separately and may be considered in specific scenarios, often alongside lifestyle measures.

#### 6.2.1 Obesity medications

For older adults with obesity whose lifestyle alone is insufficient and whose comorbidities are present, obesity pharmacotherapy might be used cautiously. Orlistat (a lipase inhibitor causing fat malabsorption) is available in many Asian–Pacific countries and can induce modest weight loss, but concerns in elderly individuals include malabsorption of fat-soluble vitamins and gastrointestinal side effects [121]. Glucagon-like peptide-1 receptor agonists (GLP-1 RAs) (such as liraglutide or semaglutide) have gained prominence as obesity treatments. They cause significant weight reduction (∼10%–15%), with a portion being lean mass (approximately 25%–30% of total weight loss) [122]. In older adults, careful monitoring of muscle mass and function is needed if GLP-1 RAs are used, as rapid weight loss without exercise might worsen sarcopenia [123]. Nonetheless, GLP-1 RAs improve physical function by reducing fat load and improving metabolic health, and small studies suggest that concurrent exercise may mitigate muscle loss [124]. A notable trial (STEP program with semaglutide) reported substantial weight loss but highlighted that approximately one-third of the weight lost was lean mass, indicating the need for mitigation strategies [125].

#### 6.2.2 Anabolic agents

No specific sarcopenia drug has been approved yet, but research is ongoing. The horizon includes potential myostatin/activin type II receptor (ActRII) inhibitors (such as bimagrumab or apitegromab), selective androgen receptor modulators (SARMs), and multimodal drugs that might target fat and muscle concurrently. Bimagrumab is a pharmaceutical agent that targets ActRII to combat obesity [126]. ActRIIs are present in adipose and myocyte cells, and upon activation, they convey signals that promote muscle degradation and diminish fat deposition [127]. Bimagrumab uniquely targets adipocytes directly without inducing appetite suppression or resulting in the loss of lean body mass, distinguishing it from most antiobesity therapies [128]. Bimagrumab is presently undergoing evaluation in a clinical trial, where it is administered either independently or in conjunction with semaglutide. The synergistic effect of GLP-1 agonists and Bimagrumab may augment fat reduction while maintaining muscle mass [129]. Testosterone replacement might be considered in older men with clinical hypogonadism and SO (low testosterone is linked to increased SO risk) [130]. However, one must weigh prostate and cardiovascular risks. SARMs such as enobosarms are under trial; early meta-analysis data suggest some increase in lean mass and fat loss, but they are not yet standard or available clinically in most places [131]. The effects of Insulin-like Growth Factor 1 (IGF-1), Growth Hormone (GH), and GH Secretagogues (GHS) supplementation in sarcopenic patients remain unstudied, emphasizing the need for future research to evaluate their potential benefits in SO [132]. Additionally, exercise mimetics such as AICAR or GW501516 may enhance muscle mass and metabolic health in SO by activating the AMPK or PPARδ pathways; however, they cannot fully replicate the comprehensive benefits of exercise on muscle strength, function, and overall health [133].

#### 6.2.3 Nutraceuticals and supplements

While not “pharmacological” in the strict sense, certain supplements are quasipharma interventions. High-dose leucine, HMB (beta-hydroxy beta-methylbutyrate, a metabolite of leucine), and creatine have been shown to support muscle preservation. HMB has been studied in sarcopenic older adults and can attenuate muscle loss, especially when HMB is combined with exercise [134]. Creatine plus resistance training helps build muscle strength and could also help older adults (provided that kidney function is okay) [135]. Asian markets often have access to these supplements, and some physicians incorporate them into management plans. Vitamin D supplementation (if deficient) is essential—considered almost a medication in deficient states because correcting it improves muscle function and reduces fall risk [95,136].

#### 6.2.4 Region-specific traditional approaches

In some Asia–Pacific countries, traditional medicine approaches are considered. For example, in India, Ayurvedic remedies (such as ashwagandha) are marketed to improve muscle strength [137]. Furthermore, traditional Chinese medicine may employ herbal tonics to stimulate Qi and strengthen muscles [109]. While evidence is limited, they may be used adjunctively by patients. Clinicians should be aware of such usage to ensure that there are no adverse interactions.

Importantly, any medication intervention must be complemented by lifestyle changes. For example, if a patient is taking GLP-1 RAs, they should also engage in strength exercises and take protein supplements; this combination strategy addresses both fat and muscle [124]. Monitoring is critical for tracking weight, BMI, muscular strength (grip, chair stands), and function to ensure that the muscle is not sacrificed unnecessarily.

## 7 Conclusions

Sarcopenic obesity is widely recognized in the Asia–Pacific region as a dual-threat illness that impacts elderly individuals. Although overall prevalence varies by region and definition, trends consistently demonstrate that it increases with age and obesity rates, indicating a growing public health burden. Sarcopenia is caused by a combination of muscle deterioration and fat buildup, which includes inflammation, hormone abnormalities, and metabolic dysregulation. Region-specific risk factors include fast lifestyle transitions (sedentary behavior, dietary changes), early-life undernutrition followed by later-life overnutrition, and cultural influences on diet and activity. Genetic predispositions and high rates of metabolic illnesses (diabetes, etc.) in Asia increase the risk.

Furthermore, screening strategies in Asia–Pacific are changing to address resource restrictions, ranging from simple calf circumference and grip strength assessments in community settings to sophisticated algorithms that incorporate AWGS sarcopenia criteria with local obesity cutoff levels. The lack of consistent criteria remains an issue, underlining the need for consensus and possibly unified Asia–Pacific rules. Nonetheless, countries such as Japan and organizations such as AWGS have established practical structures that others might follow. Additionally, lifestyle modifications are essential for management. The most common evidence-based technique for reducing fat while retaining or enhancing muscle is a combination of hypocaloric high-protein meals and regular exercise, encompassing both resistance and aerobic activities. These nonpharmacological interventions possess extensive applicability and can be customized to align with local cultures (eg, integrating traditional physical activity and dietary practices). Pharmacological alternatives are limited; weight loss drugs or hormonal therapy may be judiciously employed in certain individuals; however, none of these methods address SO adequately at this time. Consequently, prevention and early intervention through lifestyle modifications are essential.

To summarize, SO in the Asia–Pacific region represents a “convergence of 21st-century challenges”: increased longevity and rising obesity rates. Tackling this issue requires a collaborative effort across geriatrics, nutrition and fitness, modern medicine, and public health. Importantly, prevention must take precedence over treatment. By prioritizing early intervention through lifestyle modification, education, and supportive policies, we can reduce the future burden of SO. With coordinated efforts in research, clinical practice, and policymaking, it is possible to help the growing older population in the Asia–Pacific region remain healthier, be more independent, and enjoy a better quality of life. Ongoing surveillance and innovation will be essential, as this complex condition demands a holistic and culturally sensitive approach.

## CRediT author statement

**Chun-Feng Huang:** Writing – original draft, Validation. **Chih-Hsing Wu:** Visualization, Validation, Writing – review & editing.

## Conflicts of interest

The authors declare no competing interests.
